# Musculoskeletal Intervention in Children: An Update

**DOI:** 10.1007/s00270-026-04394-7

**Published:** 2026-03-24

**Authors:** Madusha Chandratilleke, Ralph Gnannt, Jared Green, Shankar Rajeswaran

**Affiliations:** 1 Department of Medical Imaging, Perth Children’s Hospital, Perth, Australia; 2 Diagnostic and Interventional Radiology, University Children’s Hospital Zurich, Zurich, Switzerland; 3 Joe DiMaggio Children’s Hospital, Envision Radiology Associates of Hollywood, 1005 Joe DiMaggio Dr, Hollywood, FL 33021 USA; 4 Department of Medical Imaging-Interventional Radiology, Ann & Robert H. Lurie Children’s Hospital of Chicago, Chicago, IL, 225 E. Chicago Avenue, Box 9, Chicago, IL USA

**Keywords:** Musculoskeletal, Ablation, Bone cyst, Pediatrics

## Abstract

This review outlines current interventional radiology literature for managing pediatric musculoskeletal conditions—specifically bone cysts, bone tumors, and pain management. With the advancements in regenerative bone grafts, ablative techniques and increased awareness from referring physicians, there has been a gradual shift from open surgical techniques to minimally invasive, image-guided procedures in the management of certain pediatric musculoskeletal conditions.

## Introduction

There is a growing body of evidence demonstrating the efficacy and safety of image-guided, minimally invasive procedures in diagnosing and treating pediatric musculoskeletal conditions. These treatment options provide safe alternatives to open surgery, particularly in disease processes known to have high recurrence rates despite open surgical techniques, or when open operative techniques are challenging or associated with significant morbidity.

The purpose of this review is to outline current interventional radiology knowledge and techniques for treatment of pediatric musculoskeletal conditions, specifically bone cysts, bone tumors and pain management and will not discuss management of soft tissue lesions such as vascular malformations. With the advancements in ablative modalities, regenerative bone grafts and increased awareness from referring physicians, musculoskeletal pathology that was previously treated with traditional surgical techniques are now amenable to minimally invasive image guided interventions.

## Unicameral Bone Cysts

Unicameral Bone Cysts (UBCs) are solitary bone lesions that are benign and of unclear etiology [1]. The true incidence of UBC in children is unknown, as there are likely many asymptomatic UBCs which are undiagnosed. UBCs primarily occur in children and adolescents, with a male predominance, and the lesions arise primarily along long bone metaphysis, specifically the femur and proximal humerus [2]. UBCs can be found incidentally on imaging or may present symptomatically with pain or fracture or decreased functionality of the affected limb [3].

There are a variety of techniques utilized to treat UBCs and a gold standard does not currently exist. Traditional minimally invasive treatment options such as cyst aspiration and injection of corticosteroid or bone marrow aspirate often requires multiple interventions, with documented recurrence rates up to 50% [4]. More invasive options such as surgical curettage and bone grafting have reported recurrence rates up to 37% [5]. Such high recurrence rates with surgical and traditional minimally invasive approaches lead some clinicians to opt for conservative treatment with observation, activity restrictions and serial imaging, as these lesions can resolve with skeletal maturity [6].

Recently, sclerotherapy has been gaining traction in treating unicameral bone cysts [7]. Disruption of the UBC wall lining has been postulated to be a critical step in arresting UBC growth, and surgeons disrupt the cyst wall with mechanical curettage. Percutaneous needle-based sclerotherapy offers a less invasive means of disrupting the UBC wall lining, functioning as a form of chemical curettage, as is widely used in visceral cysts and aneurysmal bone cysts [8]. An existing limitation of sclerosis alone is that bone regrowth across the UBC cavity often takes months to occur, which delays return to activity and requires longitudinal serial imaging to track healing, often using cross-sectional imaging [8, 9].

The development of regenerative bone graft material that can be delivered via needles has revolutionized UBC treatment. Regenerative bone grafts function to promote and expedite bone formation, which has the advantage of hastening bone healing and therefore enabling a patient to resume activity sooner [10]. Regenerative bone grafts are preferred in pediatric patients over inert agents such as polymethylmethacrylate (PMMA), as regenerative grafts resorb over time and are osteoconductive. An ideal regenerative bone graft resorbs consistently and symmetrically while promoting adjacent bone formation, as eccentric resorption can be confused with a bone cyst recurrence. There are multiple regenerative bone grafts on the market, each with varying properties and viscosities that dictate the needle gauge required to deliver the graft, which can vary between 12G and 18G. Examples of regenerative bone grafts include hydroxyapatite calcium sulfate grafts (HA-CaS04/Cerament®), calcium sulfate calcium phosphate grafts (CaSO4-CaPO4/Prodense®) and more recently magnesium phosphate grafts (Mg-PO4/Osteoinject®).

**Fig. 1:**
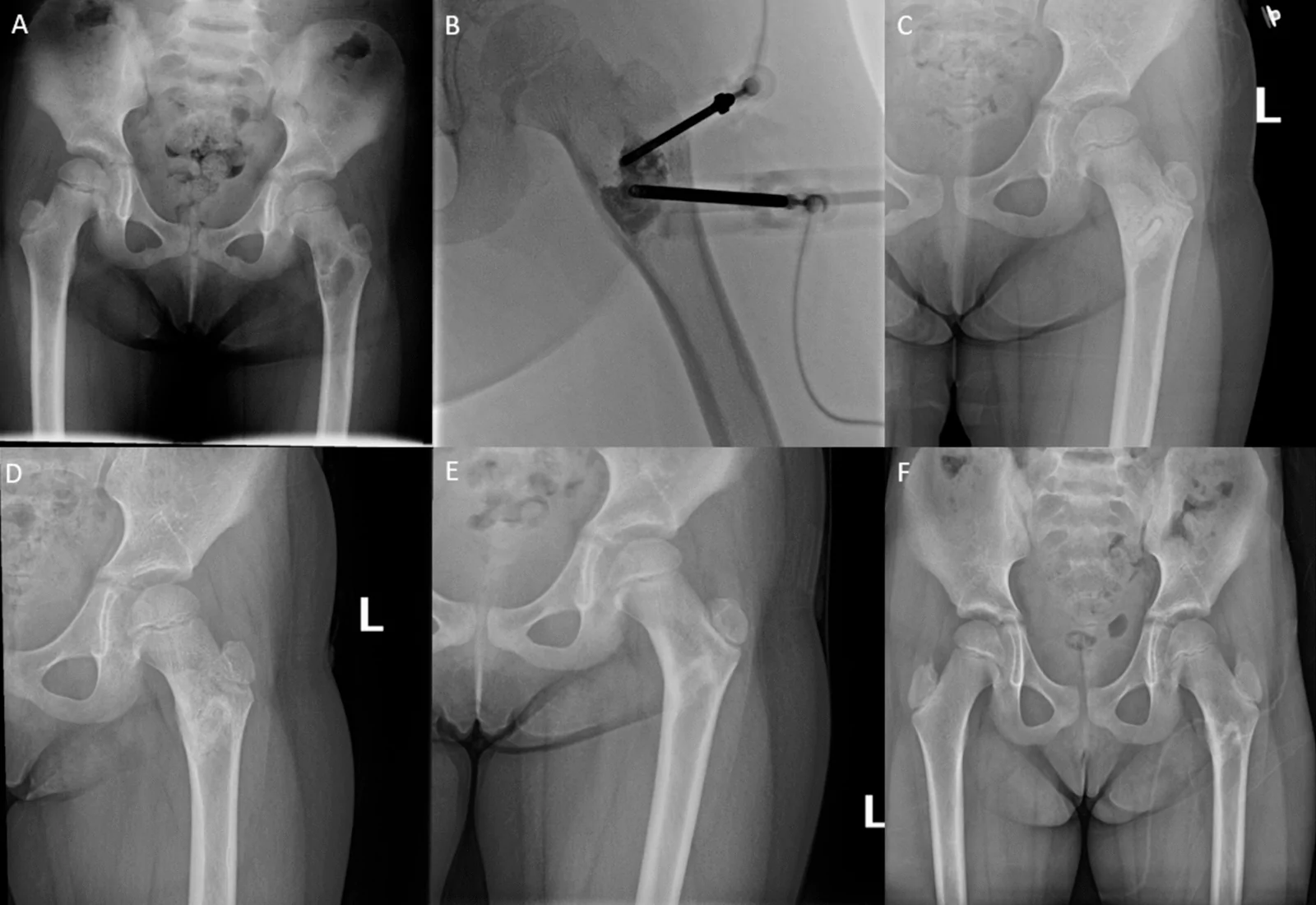
5-year-old with left hip pain. **A** Pelvic radiograph showing a unicameral bone cyst in the proximal left femur, which is traditionally managed with curettage and hardware stabilization **B** Left femur UBC treated utilizing the Sclerograft technique. **C** 3 month radiograph shows increased cortical thickening and sclerosis with partial graft resorption **D** 6 month radiograph with progressive sclerosis and near complete resorption of regenerative graft **E** 12 month radiograph with bony remodeling and increased sclerosis **F** 24 month radiograph without evidence of recurrence

**Fig. 2:**
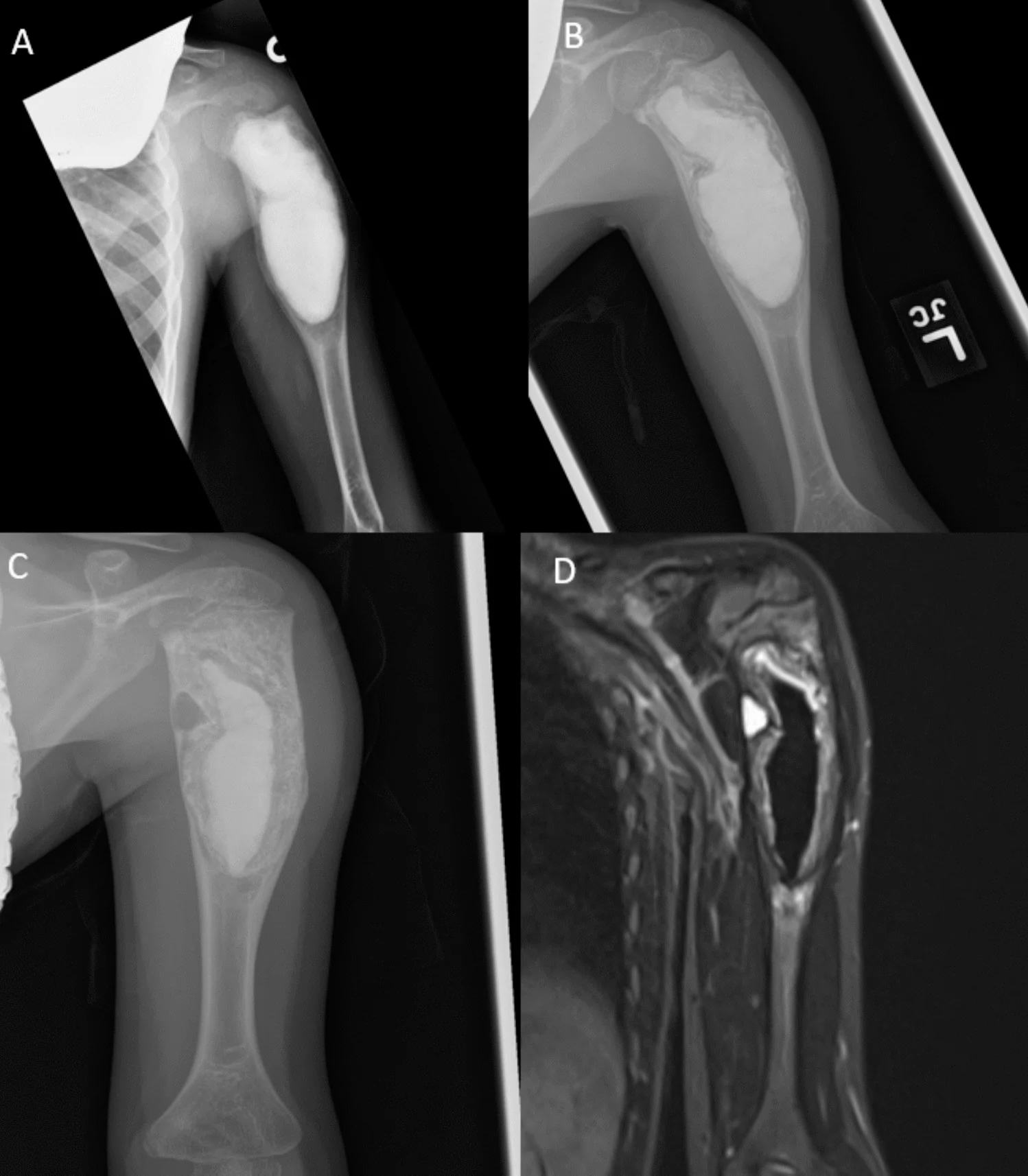
7-year-old male with history of proximal humerus UBC that underwent Sclerograft procedure. Due to filling of the defect with bone graft, recurrences can be followed with radiographs. **A** 1 month post Sclerograft with some cortical thickening **B** 3 month follow-up with some graft resorption and cortical thickening. **C** 6 month follow-up with a focal eccentric lucency, cortical thinning, and mild scalloping, consistent with a recurrence. **D** T2 STIR MRI performed to confirm recurrence which confirms the cyst recurrence along the medial wall and some fluid along the cranial aspect of the graft. Note graft on MRI STIR appears hypointense

The ability to inject regenerative bone grafts via needles into the UBC cavity, combined with chemical sclerosis, also known as the Sclerograft technique [9], shows favorable results when compared to open surgical techniques, with the added benefit of being minimally invasive. (Fig. 1). When performing Sclerograft procedures for UBC, the interventional radiologist must access the cyst in at least two positions, which is performed using ultrasound and fluoroscopic guidance. The presence of a second access needle, or venting needle, avoids the UBC from being excessively pressurized, reducing the risk of sclerosant and regenerative graft extruding beyond the cyst margins into soft tissue or egressing systemically via draining veins [9]. If sclerosant or graft leaks into the soft tissue it will cause a localized inflammatory response and pain especially if there are adjacent nerves; however, the graft will eventually resorb over months and the authors have not encountered heterotopic bone formation. An added benefit of adding regenerative bone graft with the Sclerograft procedure, in distinction to UBC treatment with sclerotherapy alone, is the improved ability to monitor cyst healing with radiographs (Fig. 2). As the previously lucent UBC cavity is now opacified with radiodense regenerative bone graft, UBC healing can often be monitored with radiographs alone, and any new areas of lucency are easily identified as cyst recurrence. This procedure is performed in a single session unlike some existing protocols for sclerotherapy alone that occur on a cadence and repeat treatment is only performed if there is radiographic evidence of recurrence.

## Aneurysmal Bone Cyst

Aneurysmal Bone Cysts (ABC) are benign but locally aggressive, lytic and expansive bone lesions and the majority occur in young patients in their first two decades of their life. Patients usually present with local pain, restricted joint function or a pathological fracture due to the thinned bone. Most ABCs occur in the long bones (63%) with the femur (22%) and tibia (17%) as the most common site followed by the spine (14%) [11, 12]. On radiographs, ABCs typically show an expansile, lytic, metaphyseal-based lesion with some periosteal reaction [12]. Magnetic Resonance Imaging (MRI) is the preferred modality to demonstrate characteristic fluid–fluid levels within cysts, enhancement of the cysts septations and to evaluate the exact extent of the lesion and possible nerve compression. 30% of ABC are secondary and may mask an underlying cause. Fast growing and solid components with contrast uptake within an ABC should raise suspicion of an underlying osteosarcoma and malignancy needs to be ruled out by the means of a biopsy [13]. Due to the risk of potential malignancy, the biopsy route should be carefully discussed with the patient’s orthopedic surgeon. Pathology findings in ABC biopsy are often nonspecific, so it is recommended to screen for a USP6 rearrangement can be found in up to 70% of ABC cases with CDH11-USP6 fusion in 30% [14].

The goal of ABC treatment is to shrink the lesion and improve bone integrity by promoting bone remodeling, reducing lesional pain and the risk of fracture. The traditional surgical approach to ABC treatment includes intralesional open curettage with bone grafting, although this approach carries a relatively high recurrence rate, even with the addition of adjunctive local therapies such as cauterization or argon beam coagulation [15]. Due to the relatively high recurrence rates of ABC managed via an open surgical approach, minimally invasive treatments have been gaining traction. Image-Guided, intralesional injection of sclerosing agents such as alcohol or doxycycline, injection of steroids, and injection of calcitonin or regenerative bone grafts are well described minimally invasive alternatives to open surgery. Comparing sclerotherapy as a treatment option to surgery alone, no statistically significant difference could be found regarding treatment success; however, functional outcomes were poorer and complications higher in the surgical group [15, 16].

An alternative treatment approach for ABC is image guided transcatheter arterial embolization, with or without a combination of percutaneous sclerotherapy. Masthoff et al. showed no recurrence of disease after a mean follow-up of 2.3 years with a mean of 1.6 transarterial embolizations and 3.2 percutaneous sclerotherapy [17]. Embolic agents include onyx, acrylic glues, particles/microspheres or coils.

**Fig. 3:**
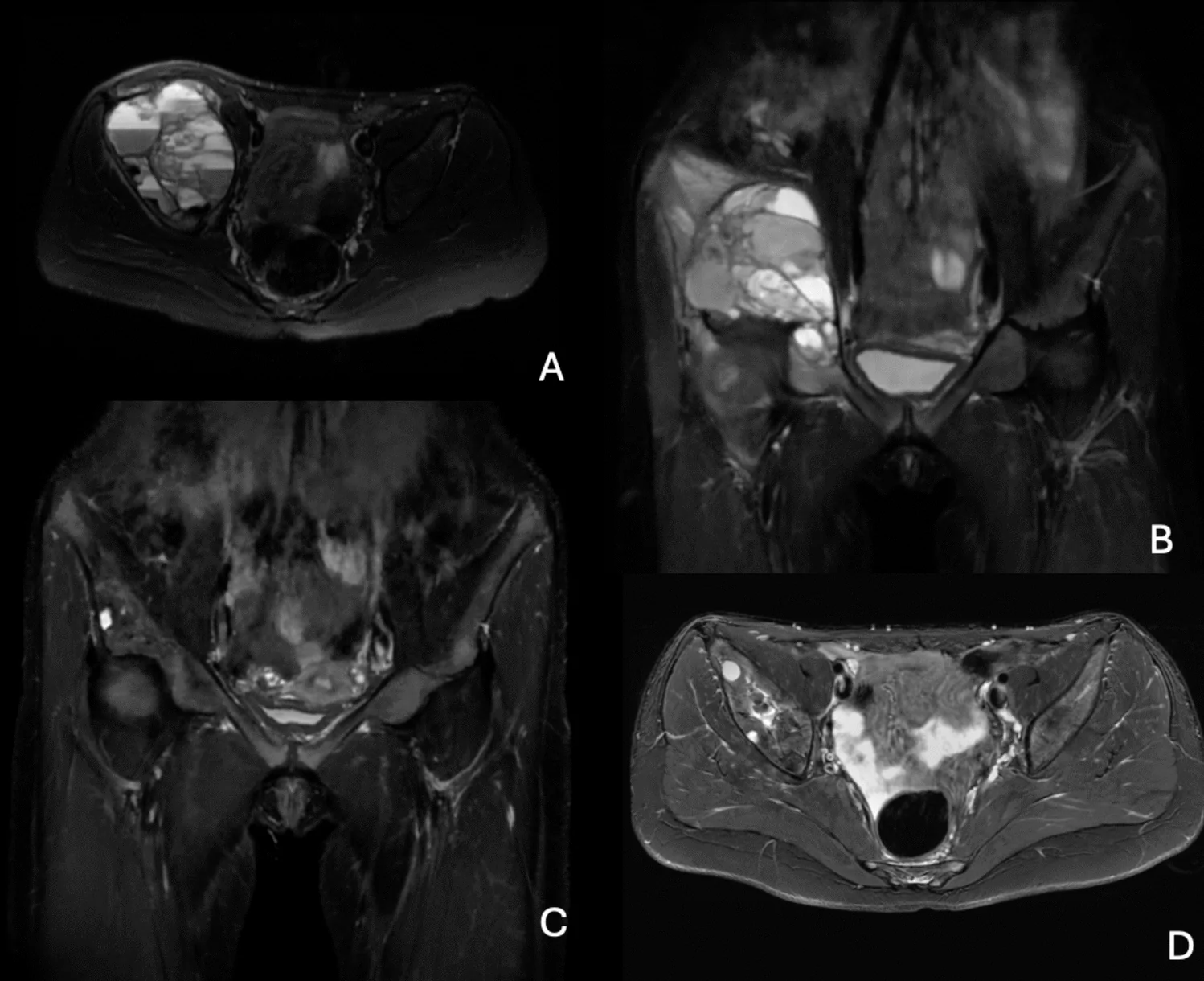
**A**&**B** 12-year-old girl with a large and painful ABC of the pelvis, axial and coronal T2 weighted MR imaging. **C**&**D** 4 years follow-up and after 2 Cryoablation sessions there is a remodeling of the bone with only minimal residual cystic lesions

Another treatment approach in ABC is cryoablation, which destroys cells through osmotic damage and necrosis and induces an immune-specific response, with necrotic bone acting as a scaffold for new bone formation [18]. Adjacent sensory nerves may be affected during ABC treatment, which can provide immediate pain relief in some cases. A recent study on ABC cryoablation described a mean number of 2 treatments with an overall lesion volume reduction of 82% at a mean follow-up of 18.3 months [19]. (Fig. 3).

A relevant concern of cryoablation in young patients is the potential for injury to an adjacent open growth plate. Cryothermal exposure of the physis may lead to premature physeal closure, resulting in limb-length discrepancy or angular deformity. Compared with intralesional curettage or sclerotherapy, cryoablation offers precise ablation control but may carry a higher risk when performed near an open physis [19]. Nerve injury is a recognized but infrequent complication of cryoablation, particularly when aneurysmal bone cysts are located in close proximity to major neural structures. Cold-induced neuropraxia may result in transient sensory and motor deficits, while permanent nerve injury appears to be rare in children. Protective strategies including hydrodissection, meticulous needle positioning real-time ice-ball visualization, temperature monitoring, and intraprocedural motor and sensory nerve monitoring may reduce the risk of neural injury and support functional recovery [20].

Conservative medical treatment of ABCs can be achieved with Denosumab, but there is still a lack of prospective, randomized studies describing efficacy of this approach. Denosumab is a monoclonal antibody interfering with maturation of osteoclasts which activates bone resorption, osteolysis and destruction in patients with ABC [21]. There are concerns about growth disturbances with Denosumab especially when treating children, although case reports do not support this assumption [22]. Further studies are needed to evaluate Denosumab as a valuable treatment option alone or in combination with more invasive procedures.

## Osteoid Osteomas

Osteoid osteomas are small benign bone tumors which have a male preponderance and most commonly present in adolescence or early adulthood [23]. Long bones and spine are the most common locations with less common sites including the skull, hand, ankle or foot [23, 24]. These tumors usually present with a classic history of night pain relieved by NSAIDS. Treatment options include medical management- namely symptom relief, percutaneous ablation, curettage or en bloc resection. Medical management has potential gastrointestinal side effects from long-term anti-inflammatory use while en bloc resection carries its own disadvantages such as risks associated with prolonged general anesthesia, difficulty locating the lesion intraoperatively, prolonged hospitalization and weakness at the resection site [25].

**Fig. 4:**
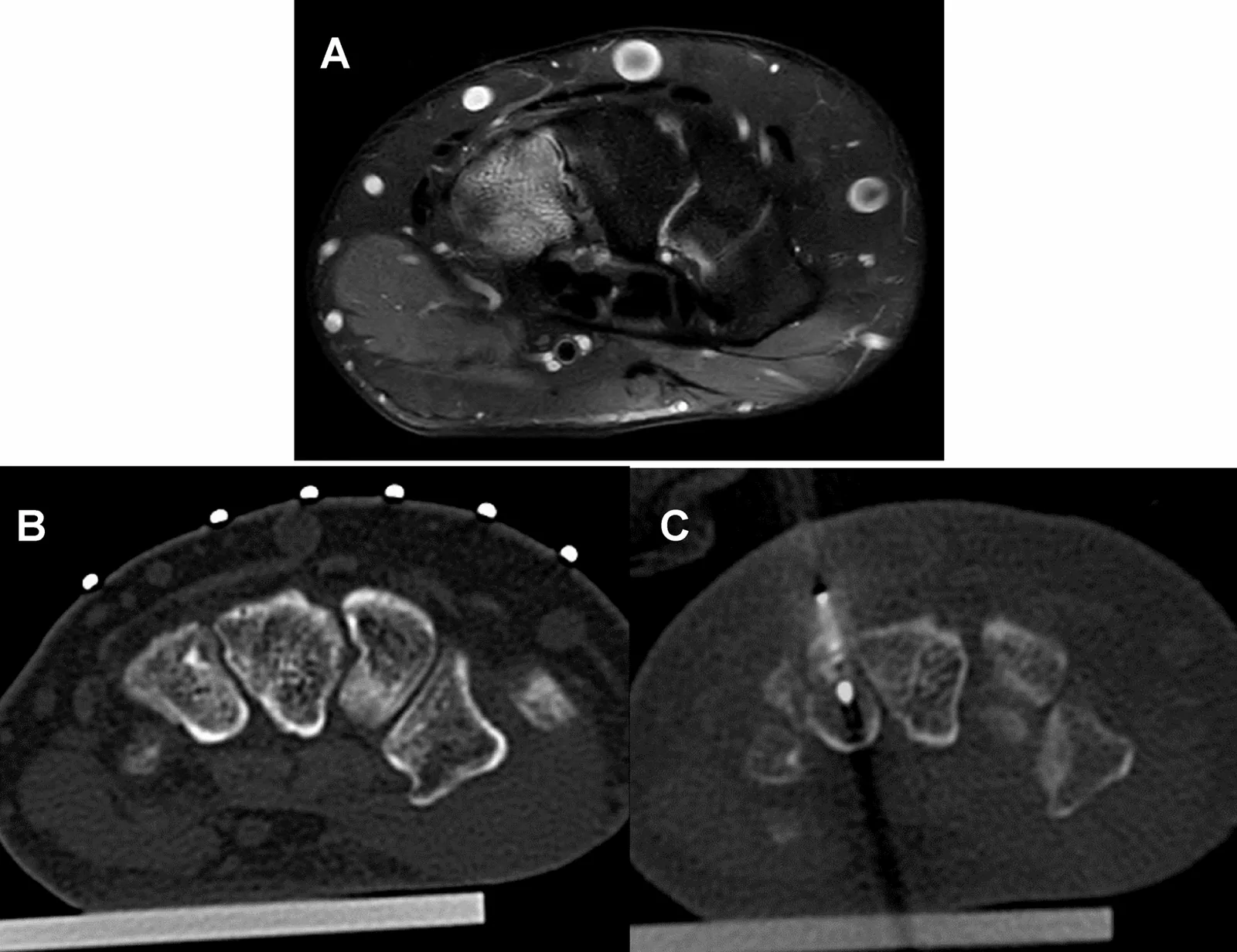
A. 16-year-old male with a Hamate Osteoid Osteoma, **B** Intraprocedural planning CT, axial through the Hamate Osteoid Osteoma. **C** Cryoablation probe in situ traversing the Osteoid Osteoma

The most widely performed percutaneous interventional treatment for an osteoid osteoma is CT guided thermal ablation, namely radiofrequency ablation (RFA), where the lesion is heated to 90 degrees celsius for 5–6 min with a 17 g RFA probe. With the more recent advent of cryoablation technology, there has been a shift toward cryoablation of osteoid osteomas (Fig. 4), with surgical intervention such as curettage and bone grafting saved for more complex cases. This shift has been particularly advantageous in the setting of tumors adjacent to vital structures such as nerves, as nerve injury from cryoablation has been shown to be temporary [26, 27]. Cryoablation involves the application of argon gas to create freeze and thaw cycles, which typically are administered for 10 min and 5 min (passive thaw) respectively. This freeze–thaw cycle is performed twice at any one probe position followed by a 2 min active thaw cycle prior to probe repositioning or removal. Interstitial Laser Ablation (ILA) is another reasonable alternative to RFA which utilizes a laser generator and optical fibers to deliver light energy and subsequent heat energy into the target treatment area. Treatment time is much shorter compared to cryoablation, requiring application of 50 degrees celsius for 30 s to achieve tissue necrosis in a 1.5 cm diameter region. Some advantages of ILA include no interference with implanted cardiac devices and reduced cost compared with other ablative techniques, with comparable success rates [28].) However, the optical fibers are not radiopaque and therefore careful measurement of the fibers and guiding coaxial system must be done before procedure commencement to ensure adequate fiber placement and subsequent ablation. Any of the above forms of ablation typically start with percutaneous CT guided bone drilling, using either the Arrow Oncontrol 11 g/15 g battery powered drill or Bonopty 14 g manual drill, into the nidus of the lesion, which on its own has been shown to have successful outcomes in over 80% of patients [27, 29].

An innovative, emerging ablation technique which does not require bone drilling is Magnetic Resonance guided Focused Ultrasound (MRg-FUS). MRg-FUS is a completely non-invasive technique which harnesses the conversion of mechanical energy into heat, focused transcutaneously on a small tissue volume, without incisions, ablating the lesion and creating periosteal neurolysis and associated pain relief [30, 31]. This therapeutic modality uses detailed MRI for treatment planning and MRI thermometry for real-time therapy monitoring and control.

The major advantages of this technique in the pediatric setting are the total absence of ionizing radiation and non-invasive nature. No bone biopsy is taken and therefore the patients most suitable for MRg-FUS are those in which there is little doubt regarding the diagnosis. Additionally, there should be no general contraindication for MRI. Reported success rates of this technique are as high as 97% [32]. Limitations of this method include the following: the lesion must be 'acoustically accessible,’ meaning there is no overlying bowel gas, metal or scars, procedure time and the patient must be positioned so that the area to be treated is located within the accessible zone. Varying success rates of each ablation method are reported among the literature, and many studies compare all ablation techniques as a group vs surgical resection. A summary table of the individual success rates, advantages and disadvantages are listed in Table 1 [33–39]. Microwave ablation has been omitted as there is comparatively limited data on treatment of osteoid osteoma compared with other ablation techniques and this technique is typically suited to larger lesions.

**Table 1:** Osteoid Osteoma Treatment Comparison

	Resection	RFA	Cryoablation	ILA	MRg-FUS
Success rate	~ 61%	> 96%	> 90%	> 94%	> 90%
Recurrence	4.5–25%	~ 6% but reported as low as 2%	~ 1%	~ 5%	None reported, but small sample sizes and little long term information in literature
Advantages	Complete lesion removal in most cases, therefore greater chance of a definitive pathology result	Short procedure time	Lower risk of permanent damage to neural structures Ice ball visualization (within soft tissues) Flexible size of ablation zone	Does not require neutral electrodes or current through the patient More cost effective compared to RFA Short procedure time	Non invasive No ionizing radiation
Disadvantages	Higher complication rate Higher recurrence rate if incompletely excised Longer anesthetic/ procedural and recovery time No longer the gold standard	Permanent damage to neural structures Large nidus > 10 mm requires multiple probes	Long procedure time	Fibers are not radiopaque and can break	Limited access to equipment Limited procedural familiarity No biopsy Limited use depending on tumor location—best for non-spinal OOs

## Bone Metastases

Optimal treatment planning of metastases requires accurate evaluation on both CT and MR. Unenhanced CT is essential, as it allows the characterization between osteoblastic and osteolytic metastases and detects integrity or infiltration of adjacent cortical bone. In contrast, MRI provides superior delineation of the metastasis borders and its characteristics in terms of cellular density, vascularization, etc. These parameters are fundamental for the evaluation of treatment outcomes at follow-up [39].

In addition to RFA and cryoablation, microwave ablation (MWA) and Focussed Ultrasound have also been utilized successfully in the treatment of bone metastases. MWA utilizes 13–17 g probes, delivers higher heat quickly and has the advantage of being able to treat larger tumors up to 6 cm compared with RFA, which is limited to treatment of smaller tumors around 3 cm in size. MWA also reports less heat sink effect compared to RFA. Cryoablation and MWA both have theoretical advantages over RFA in energy conduction through cortical or very sclerotic bone as the effectiveness of RFA relies on the presence of water molecules in the lesion [40]. The largest ablation zone can be produced by cryoablation by utilizing multiple probes in various orientations with the downside of having longer procedural and anesthetic time compared with other ablation techniques.

As described in the previous section, Focussed Ultrasound does not require a skin incision, therefore this therapy should be utilized in patients who do not require a biopsy for pathology confirmation, those who already have an established diagnosis from a prior biopsy or those who require palliative symptom relief [41]. In cases which require pathology confirmation and therefore biopsy, alternate ablative therapies should be selected [39].

## Other Primary Bone Tumors

**Fig. 5:**
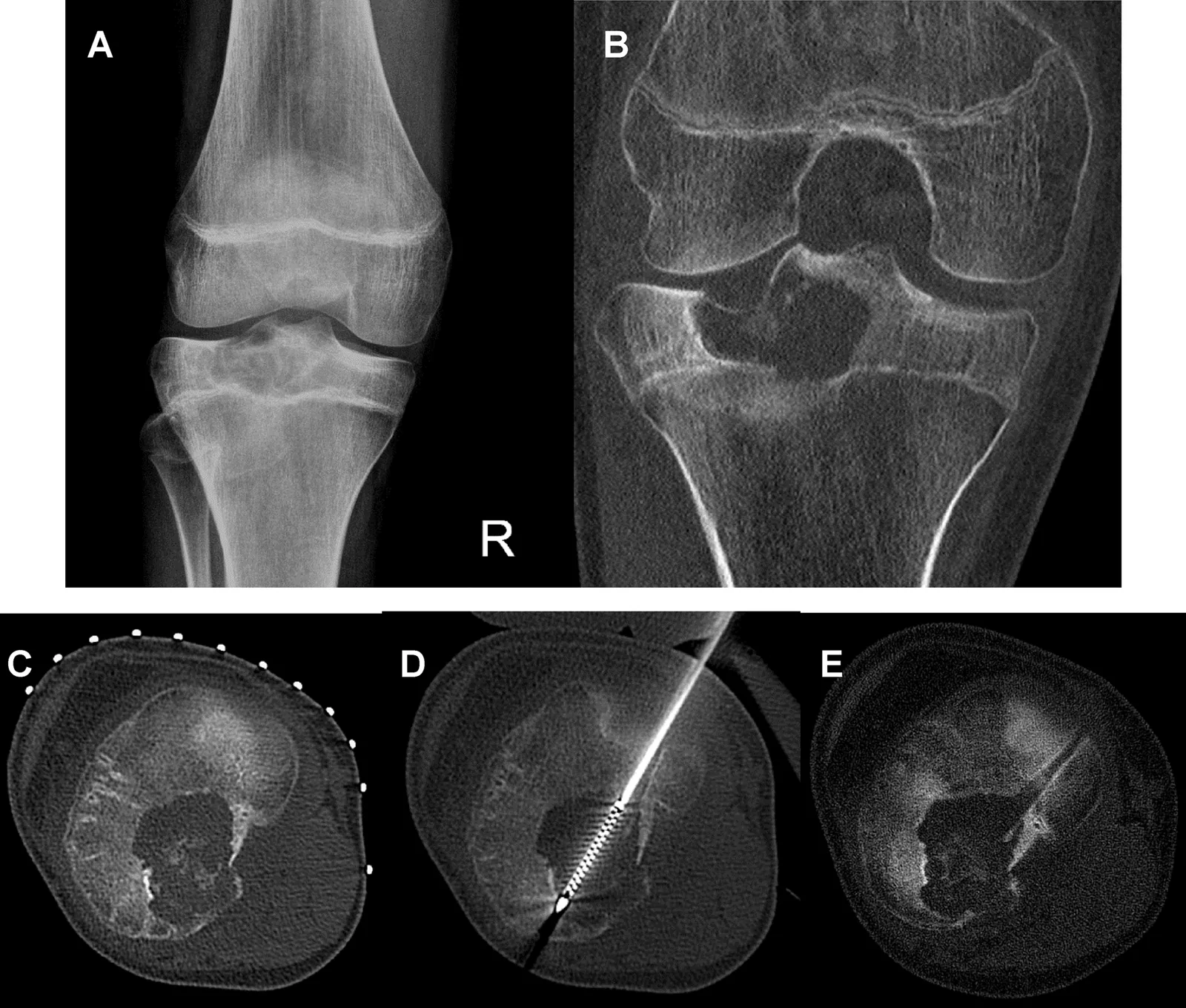
**A** Preprocedure right knee radiograph demonstrating a lucent lesion in the proximal tibial epiphysis. **B** Preprocedure right knee CT demonstrating a lucent lesion in the proximal tibial epiphysis. **C** Intraprocedural planning CT, axial through the lesion. **D** Cryoablation probe in situ traversing the lesion. **E** 6 weeks post cryoablation demonstrating early sclerosis surrounding the lesion. No bony ingrowth at the site of the lesion. Biopsy proven chondroblastoma

Management of primary tumors such as chondroblastoma/osteoblastoma often require additional considerations such as potential physeal damage and preservation of the integrity of any weight bearing bones. The mainstay of treatment has been surgical curettage followed by bone grafting however percutaneous cryoablation as an alternate therapy has the distinct advantages of shorter recovery time, less postoperative pain and less damage to surrounding structures [42]. (Fig. 5) Surgical data has shown that combining cryosurgery with bone grafting may be the optimal approach to achieving tumor control and bone integrity [43]. Extrapolating from this method and adapting it to Interventional Radiology, a possible future direction may include initially performing a biopsy and cryoablation of the lesion, followed by regenerative bone graft injection with imaging guidance at a future date if there is concern for bone integrity.

## Pain Management

Nerves are considered by many pediatric interventional radiologists as a structure to avoid during procedures, but intentional image guided treatment of nerves can be performed as an adjunct to a painful procedure, or as a standalone procedure to address pain. Chronic pain is an important issue in pediatrics, as it can greatly impact overall functioning and quality of life, and has even been shown to impact school performance [44].

Nerve blocks are performed under imaging guidance to deliver local anesthetics, sometimes mixed with steroids or other adjuvants, in proximity of the desired peripheral nerve. Care must be taken to avoid local anesthesia systemic toxicity in pediatric patients by paying close attention to the mg/kg dose maximum for each medication [45]. Nerve blocks have been shown to reduce intraprocedural and post procedure narcotic use, improve patient pain scores, and shorten post procedure length of stay [45, 46].

A recent publication has shown the value of performing a nerve block in pediatric patients prior to painful extremity IR procedures, including percutaneous treatment of bone cysts, in order to reduce post procedure pain and improve patient satisfaction [46]. The authors described an 86% reduction in intraprocedural opioid usage and 83% reduction in total post procedure opioid usage among the cohort [46]. Additionally, average maximum pain scores in PACU were significantly reduced, as was the post procedure recovery time in bone cyst patients [46].

Nerve blocks can also be used to diagnose and treat certain musculoskeletal conditions in pediatric patients. Chronic abdominal wall pain has been described in up to 10–19% of children, and anterior cutaneous nerve entrapment syndrome (ACNES) is one of the potential causes of such pain [47]. ACNES is likely under-recognized as a cause of anterior abdominal wall pain, and most patients have no history of trauma or prior surgery [48]. The abdominal cutaneous nerve arises from the T7-12 intercostal nerves. The anterior branch of the abdominal cutaneous nerve turns 90° as it crosses the rectus muscle fascia, and compression of the nerve at this point, especially during abdominal wall muscle contraction, is thought to cause nerve irritation and ischemia responsible for ACNES pain [48].

**Fig. 6:**
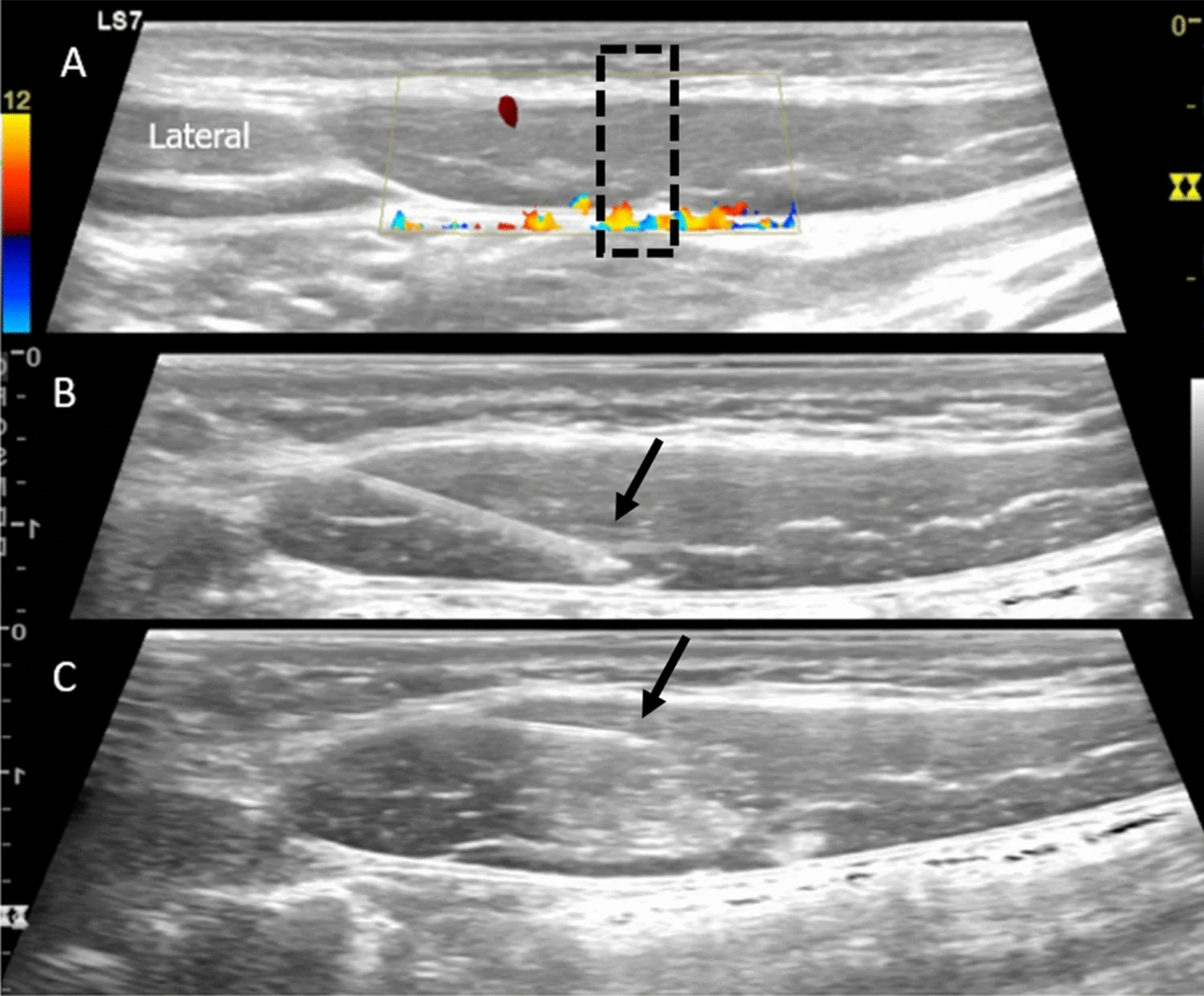
12-year-old female with Anterior Cutaneous Nerve Entrapment Syndrome (ACNES). **A** Sonographic image showing the rectus muscle and the location of anterior cutaneous nerves as it exists laterally (dashed box) **B** 25 g needle (arrow) positioned deep within the muscle and a combination of ethanol and Marcaine 1% was injected **C** Needle (arrow) positioned more superiorly to chemically ablate the nerve along its entirety

Ultrasound guided nerve blocks or trigger point injections can be diagnostic of ACNES and provide therapeutic benefit. A meta analysis of pediatric patients undergoing trigger point injections with local anesthesia, with or without steroids, showed reduced pain or resolved pain in 38–87% of patients [47]. Ultrasound guidance during injections is recommended to ensure medication delivery to the correct anatomic space as well as to avoid peritoneal puncture [49]. Patients who respond to ultrasound guided trigger point injections but have recurrent pain can pursue more durable treatment with thermal ablation, ethanol neurolysis or surgical anterior neurectomy [47, 50] (Fig. 6).

Another musculoskeletal condition which may benefit from an image guided nerve block is slipping rib syndrome. Slipping rib syndrome results from hypermobile false ribs anteriorly (ribs 8–10) or floating ribs (ribs 11 and 12), which can slip beneath, or abut, the rib above, causing intercostal nerve impingement and pain [51]. Nerve blocks can be used to localize the affected level and provide temporary pain relief, although there are reported cases of durable permanent pain relief as well [52]. The mainstay of slipping rib syndrome therapy is cartilaginous rib excision, and pediatric IRs can contribute to the care of these patients by offering preoperative intercostal nerve cryoneurolysis.

**Fig. 7:**
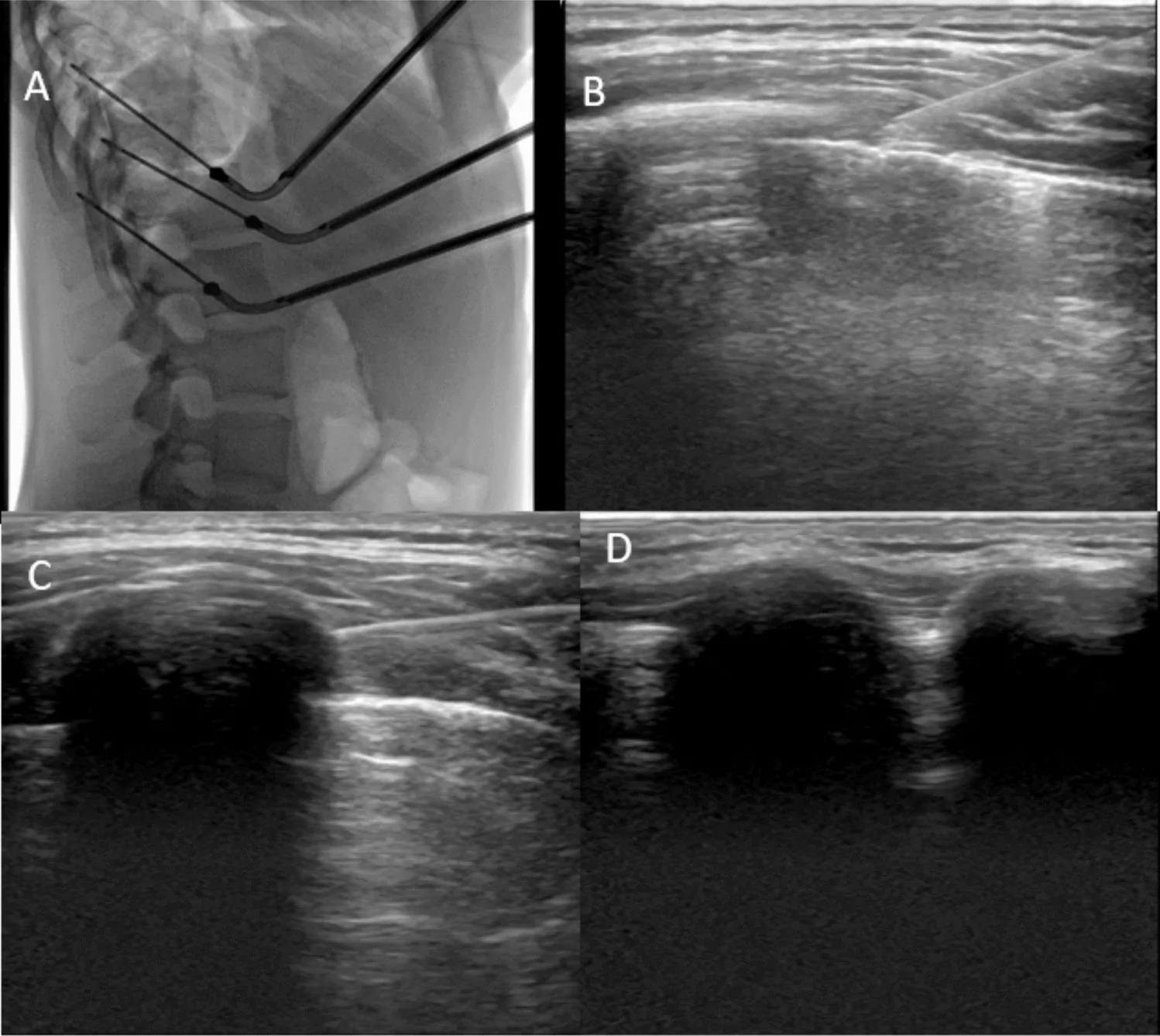
12-year-old female with clinical history suggestive of slipping rib syndrome. **A** Fluoroscopic image showing 3 cryoablation probes below right sides ribs 9–11. **B** Longitudinal sonographic image showing the probe abutting the pleural surface **C**. Longitudinal sonographic image showing formation of an ice ball at the intercostal space **D** Transverse sonographic image showing adjacent ice balls and multiple intercostal levels

Cryoneurolysis is a minimally invasive technique which exposes nerves to ice crystals causing loss of function and Wallerian degeneration with intention of relieving or preventing pain [53]. Intercostal cyroneurolysis can be performed preoperatively or intraoperatively for pectus repair, slipping rib repair or rib resection and thoracotomy for other indications and has been shown to provide decreased opioid use post op and decreased length of stay [52]. Interventionalists must be cautious when performing intercostal cryoablation below the ninth rib, as these intercostal nerves contribute to abdominal wall motor innervation and may lead to weakness of abdominal muscles and abdominal wall laxity [52]. Two series describing intercostal nerve cryoablation in pediatric patients series of cryoablation for cartilaginous rib excision showed no incidence of abdominal wall laxity post cryo despite treating below T9 [51, 54]. Any motor nerve deficits after cryoablation can be expected to recover, with nerve function returning at an average of 1.5 mm per day, ranging from less than 0.3 mm/day to 4.1 mm/day [55] (Fig. 7).

In conclusion, there has been significant development in novel techniques used to treat pediatric musculoskeletal conditions in recent years. With overall lower morbidity and often comparable efficacy to traditional surgical approaches, interventional radiology plays a critical role in managing pediatric musculoskeletal conditions.
